# Supplementary figures and images for: Rapid cloning-free mutagenesis of new SARS-CoV-2 variants using a novel reverse genetics platform
Source: eLife. 2023 Nov 21;12:RP89035. doi: 10.7554/eLife.89035 (PMC10662946; doi:10.7554/eLife.89035)

Figure 1

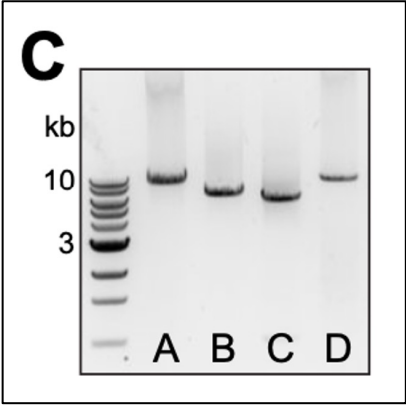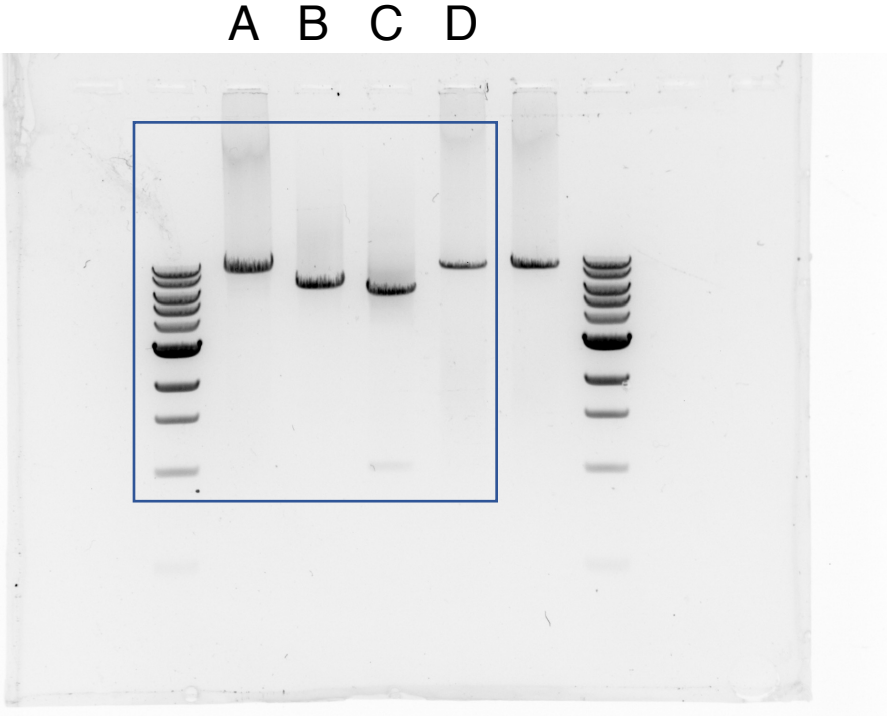

Supplement: Figure 1—source data 1. [file elife-89035-fig1-data1.zip › Figure 1-Source Data 1/Figure 1-Source Data 1.pdf]

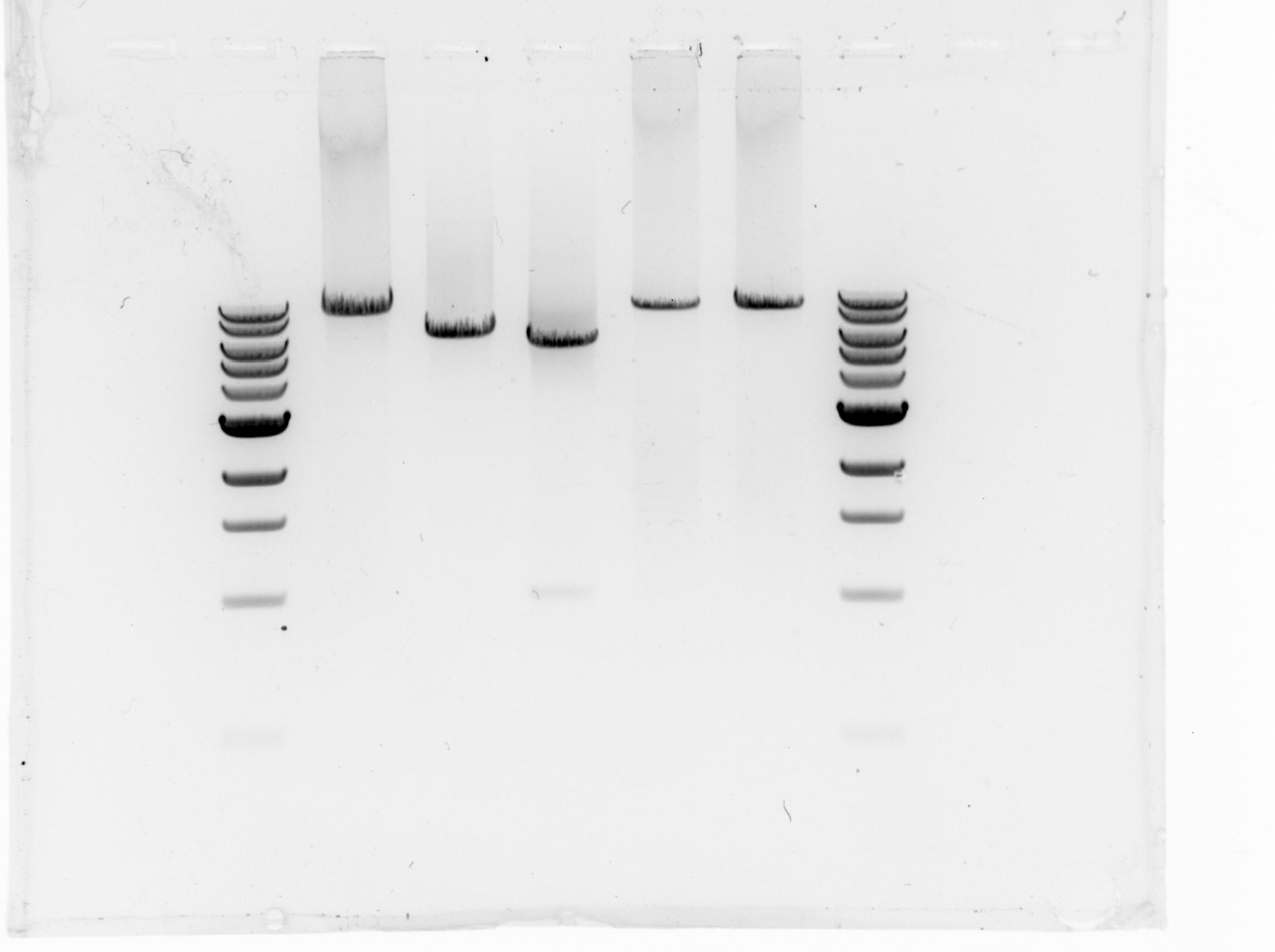

Supplement: Figure 1—source data 1. [file elife-89035-fig1-data1.zip › Figure 1-Source Data 1/Figure 1C_gel uncropped.tif]

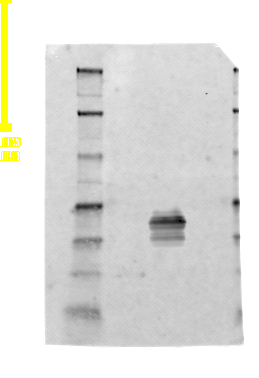

Supplement: Figure 4—source data 1. [file elife-89035-fig4-data1.zip › Figure 4-Source Data 1/Figure 4C_blot uncropped_a-ORF3a.tif]

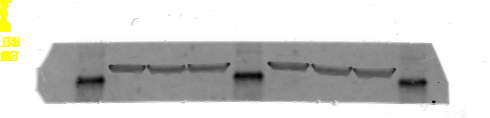

Supplement: Figure 4—source data 1. [file elife-89035-fig4-data1.zip › Figure 4-Source Data 1/Figure 4C_blot uncropped_a-b-ACT_2.tif]

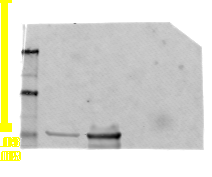

Supplement: Figure 4—source data 1. [file elife-89035-fig4-data1.zip › Figure 4-Source Data 1/Figure 4C_blot uncropped_a-NSP2_1.tif]

Figure 4

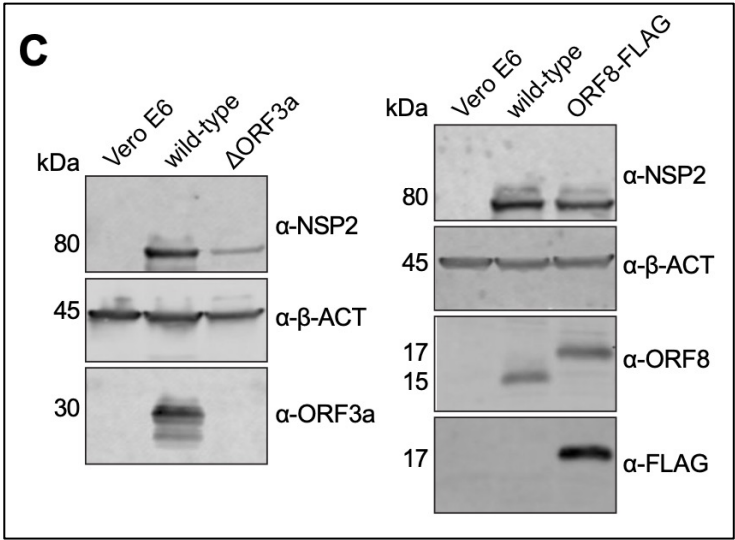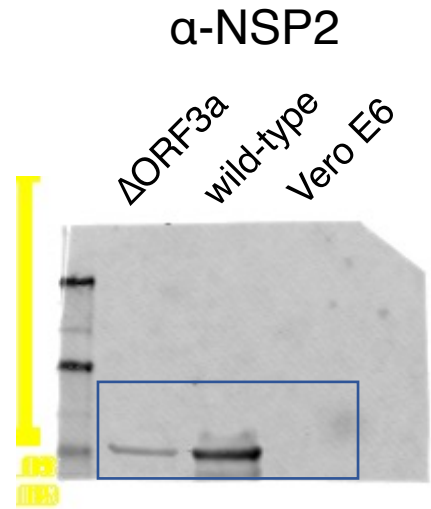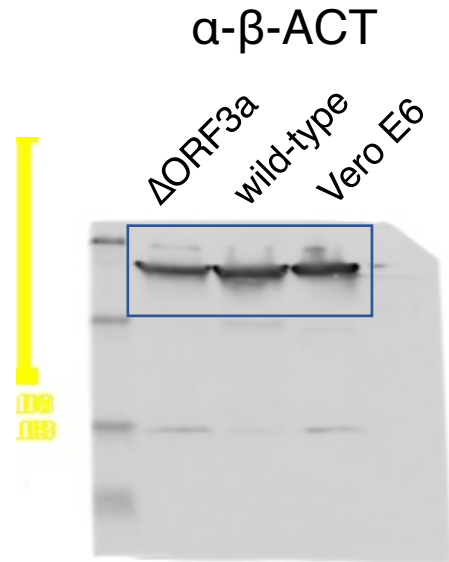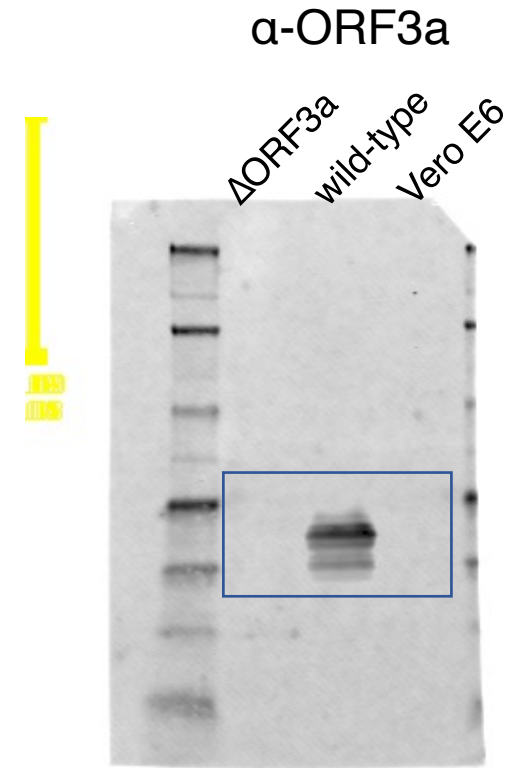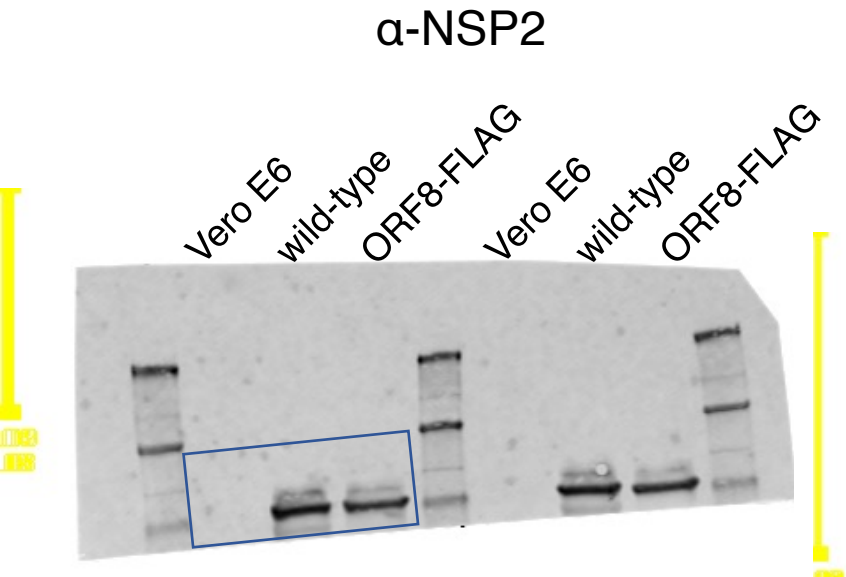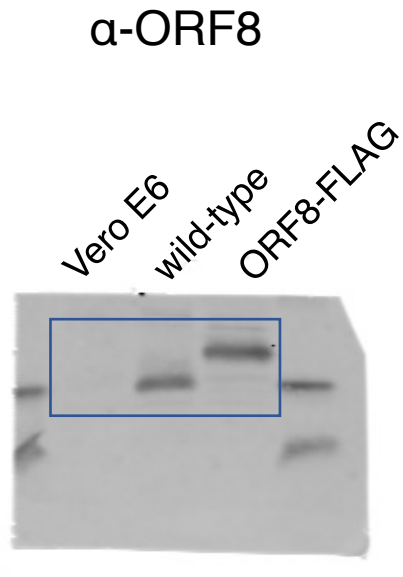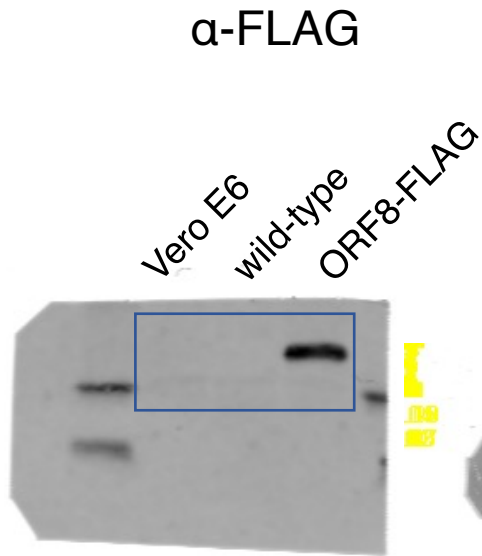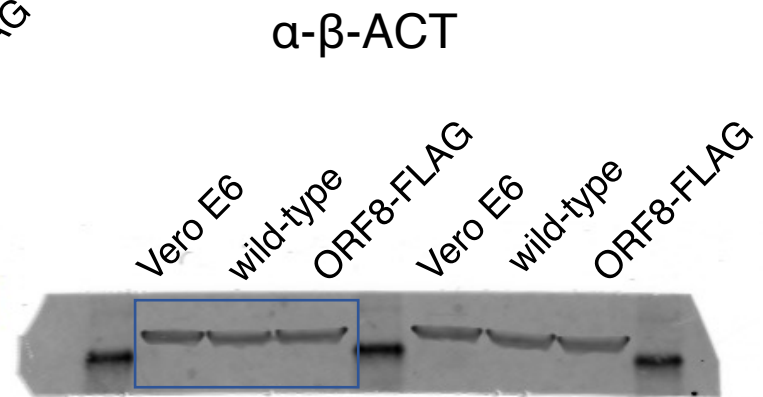

Supplement: Figure 4—source data 1. [file elife-89035-fig4-data1.zip › Figure 4-Source Data 1/Figure 4-Source Data 1.pdf]

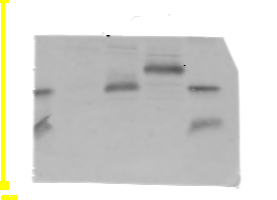

Supplement: Figure 4—source data 1. [file elife-89035-fig4-data1.zip › Figure 4-Source Data 1/Figure 4C_blot uncropped_a-ORF8.tif]

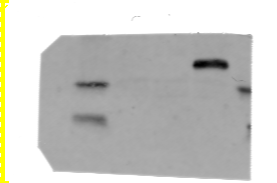

Supplement: Figure 4—source data 1. [file elife-89035-fig4-data1.zip › Figure 4-Source Data 1/Figure 4C_blot uncropped_a-FLAG.tif]

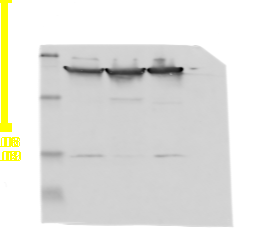

Supplement: Figure 4—source data 1. [file elife-89035-fig4-data1.zip › Figure 4-Source Data 1/Figure 4C_blot uncropped_a-b-ACT_1.tif]

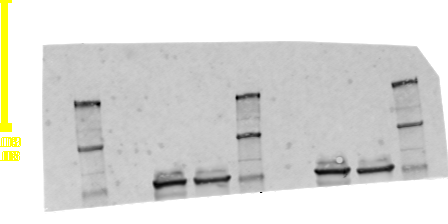

Supplement: Figure 4—source data 1. [file elife-89035-fig4-data1.zip › Figure 4-Source Data 1/Figure 4C_blot uncropped_a-NSP2_2.tif]

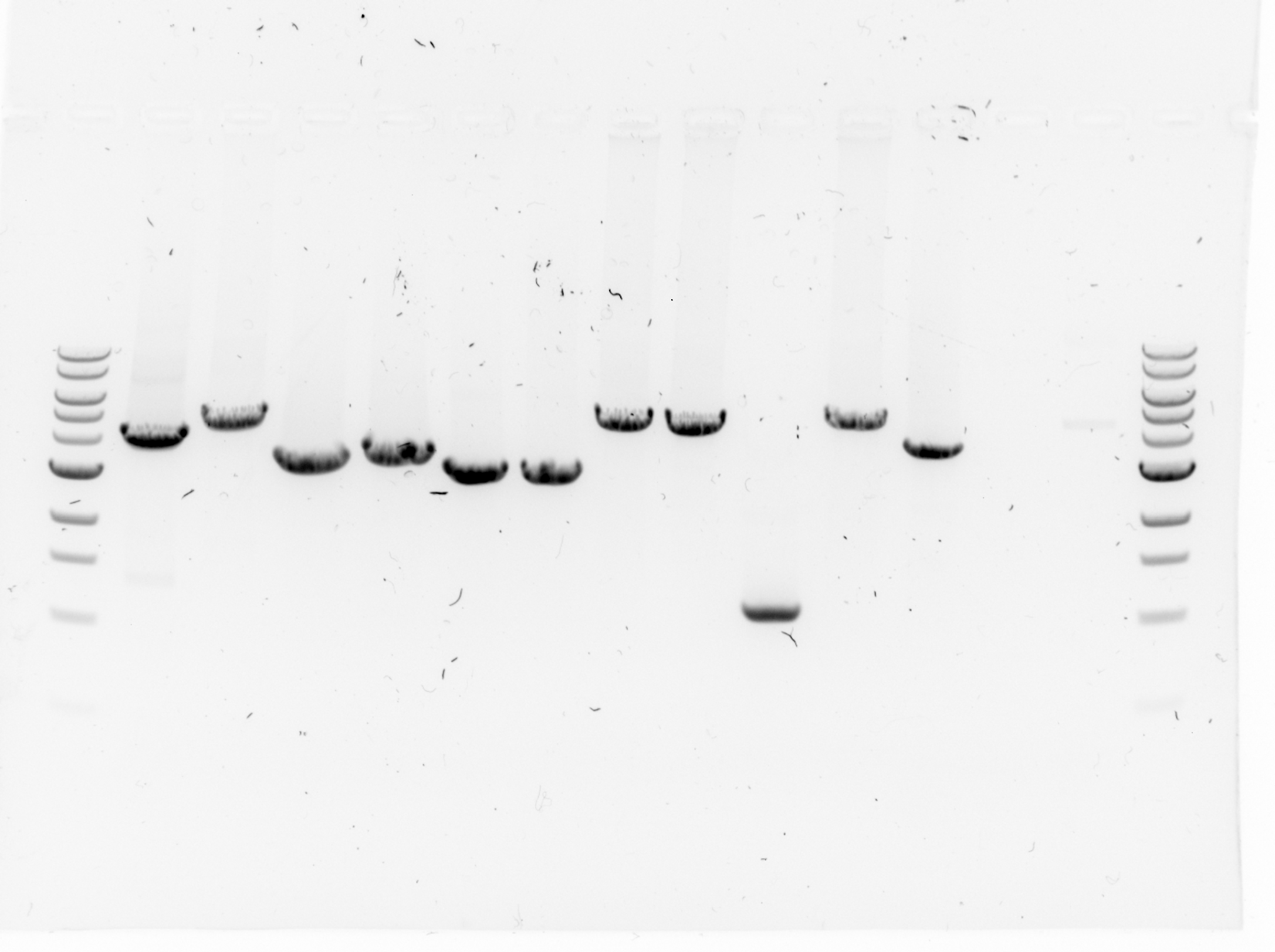

Supplement: Figure 5—source data 1. [file elife-89035-fig5-data1.zip › Figure 5-Source Data 1/Figure 5B_gel uncropped_3.tif]

Figure 5

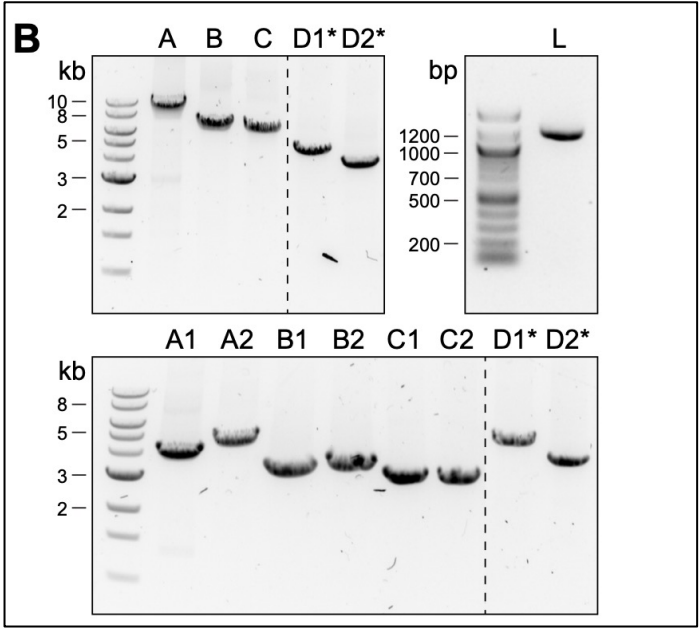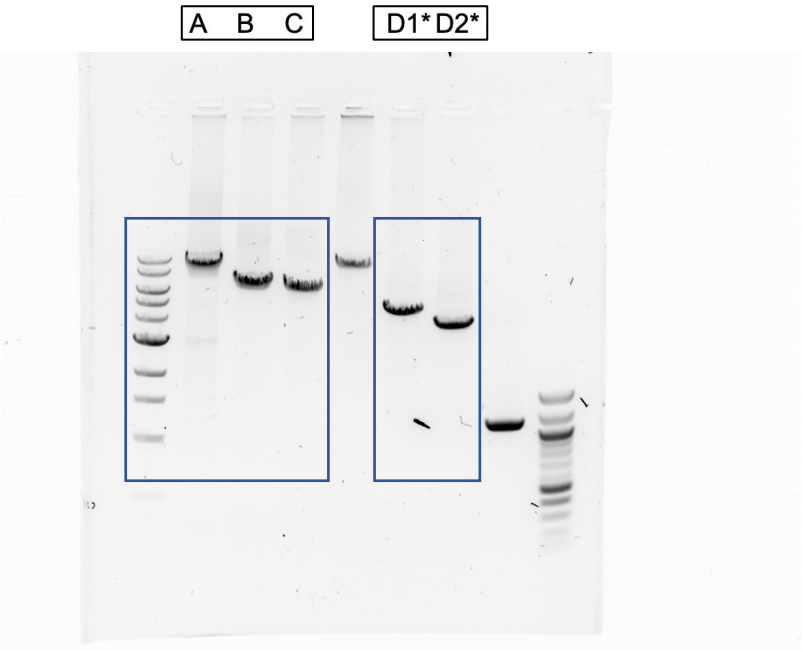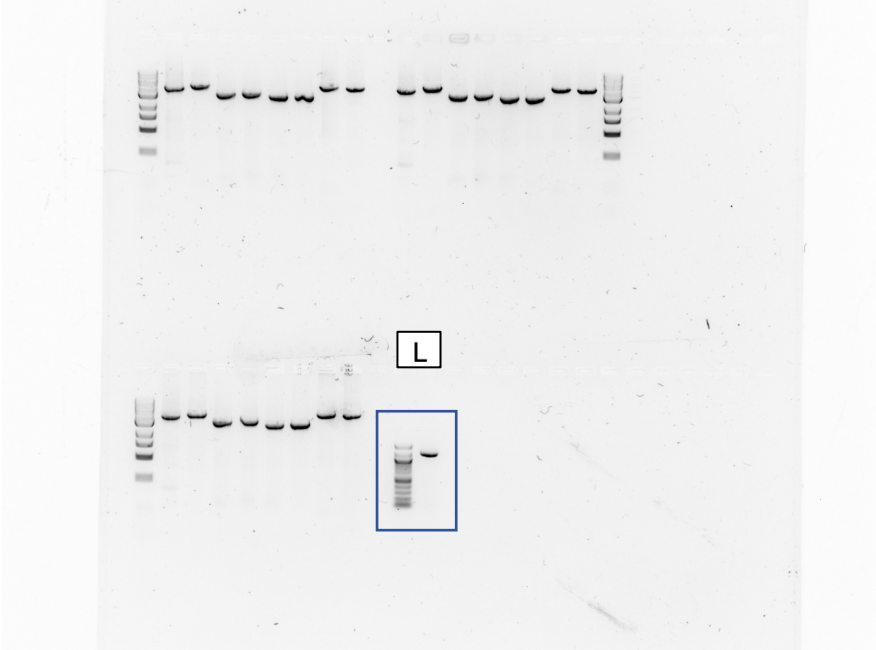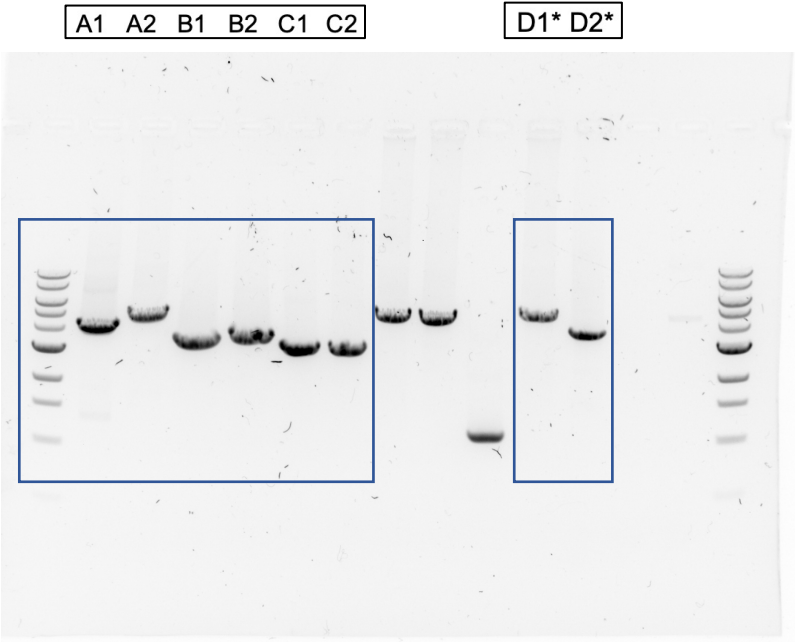

Supplement: Figure 5—source data 1. [file elife-89035-fig5-data1.zip › Figure 5-Source Data 1/Figure 5-Source Data 1.pdf]

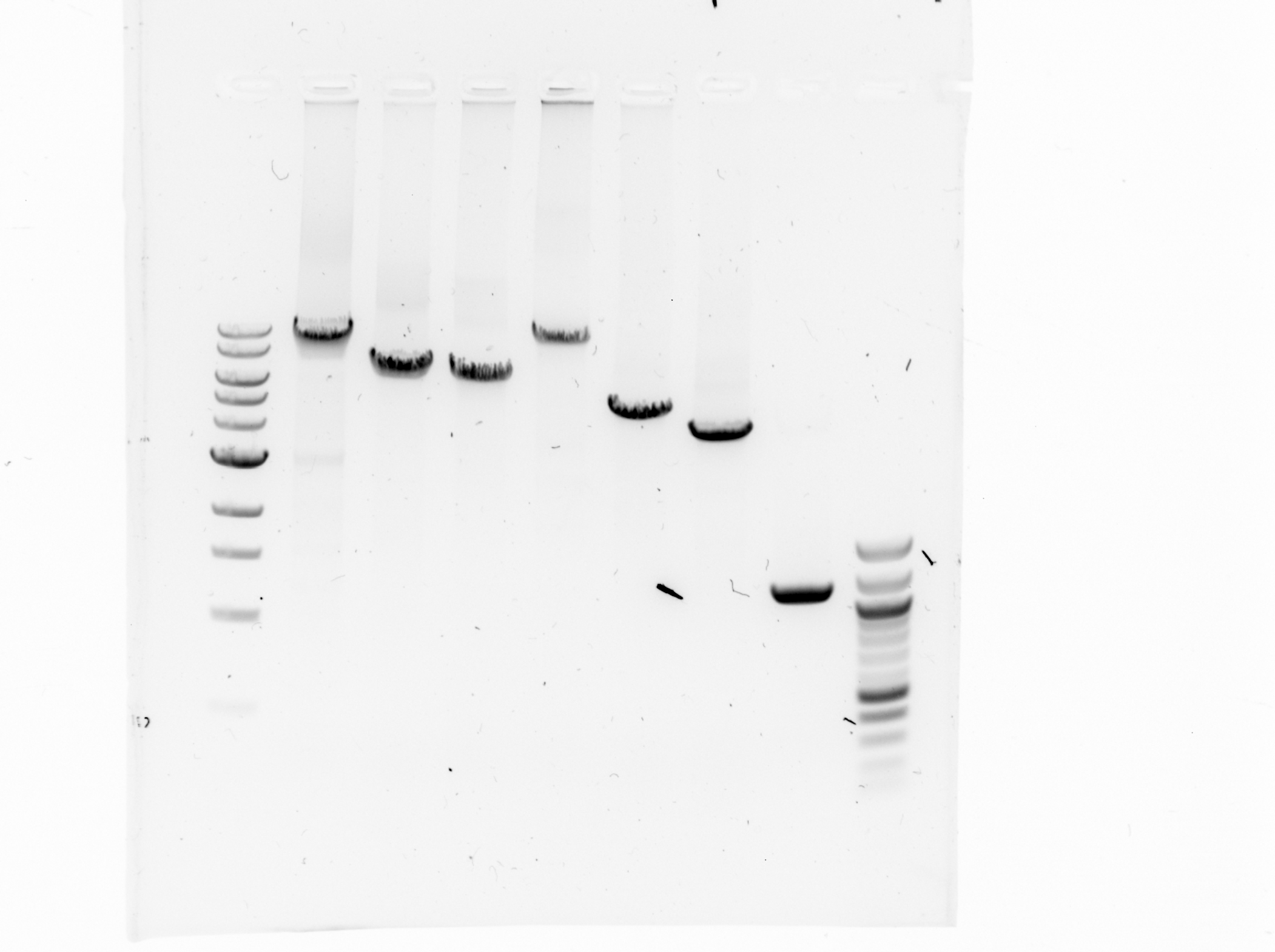

Supplement: Figure 5—source data 1. [file elife-89035-fig5-data1.zip › Figure 5-Source Data 1/Figure 5B_gel uncropped_1.tif]

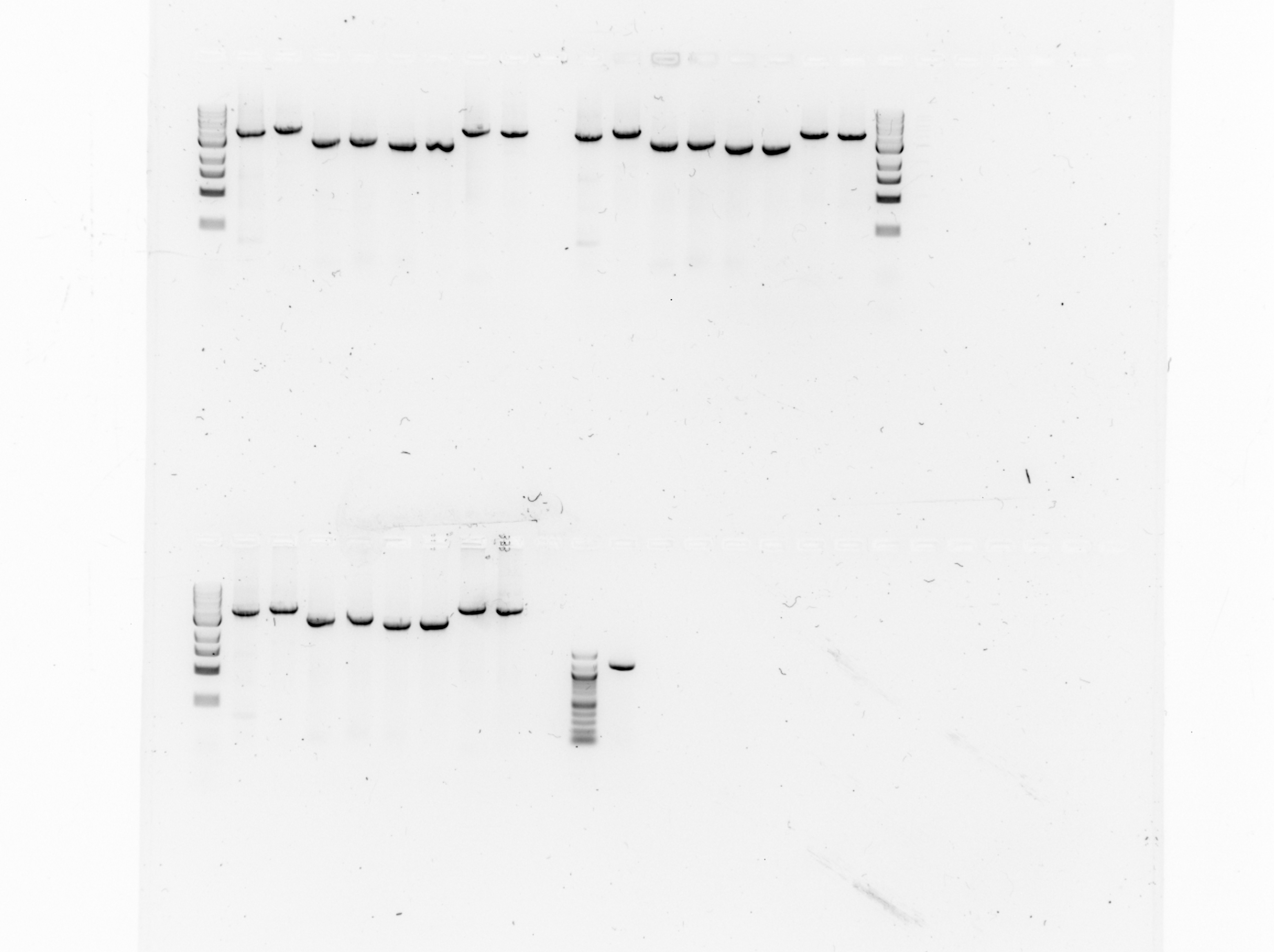

Supplement: Figure 5—source data 1. [file elife-89035-fig5-data1.zip › Figure 5-Source Data 1/Figure 5B_gel uncropped_2.tif]

Figure 5-figure supplement 1

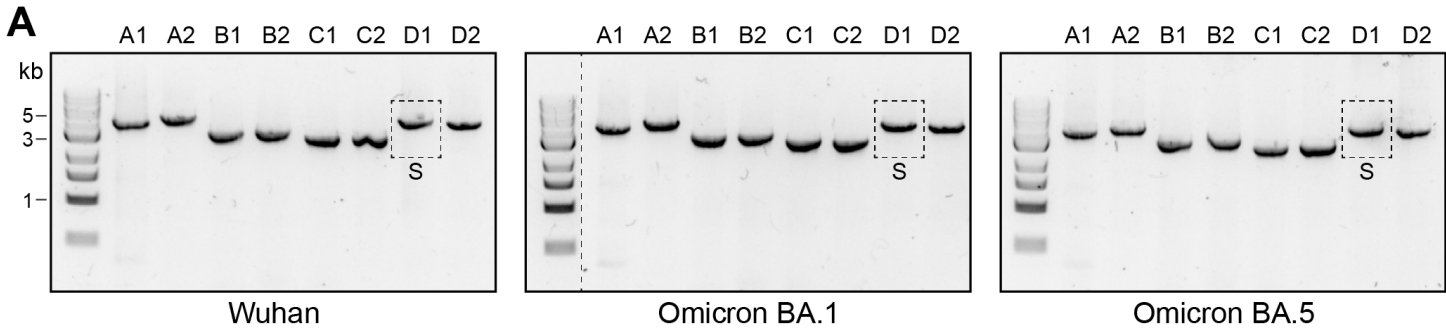

Wuhan Omicron BA.1

A1 A2 B1 B2 C1 C2 D1 D2 A1 A2 B1 B2 C1 C2 D1 D2

Omicron BA.5

A1 A2 B1 B2 C1 C2 D1 D2

Supplement: Figure 5—figure supplement 1—source data 1. [file elife-89035-fig5-figsupp1-data1.zip › Figure 5-figure supplement 1-Source Data 1/Figure 5-figure supplement 1-Source Data 1.pdf]

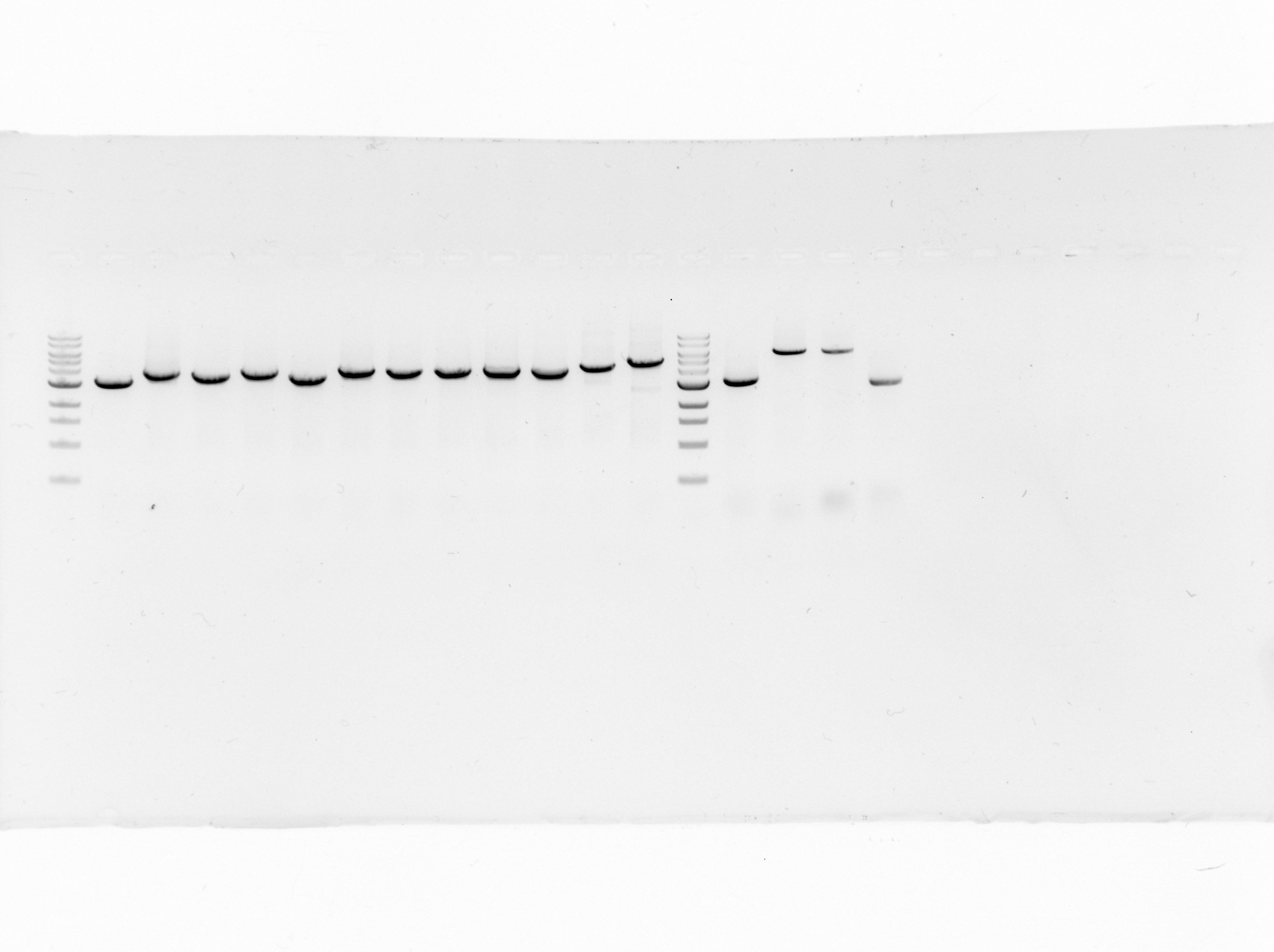

Supplement: Figure 5—figure supplement 2—source data 1. [file elife-89035-fig5-figsupp2-data1.zip › Figure 5-figure supplement 2-Source Data 1/Figure 5-figure supplement 2B_gel uncropped_1.tif]

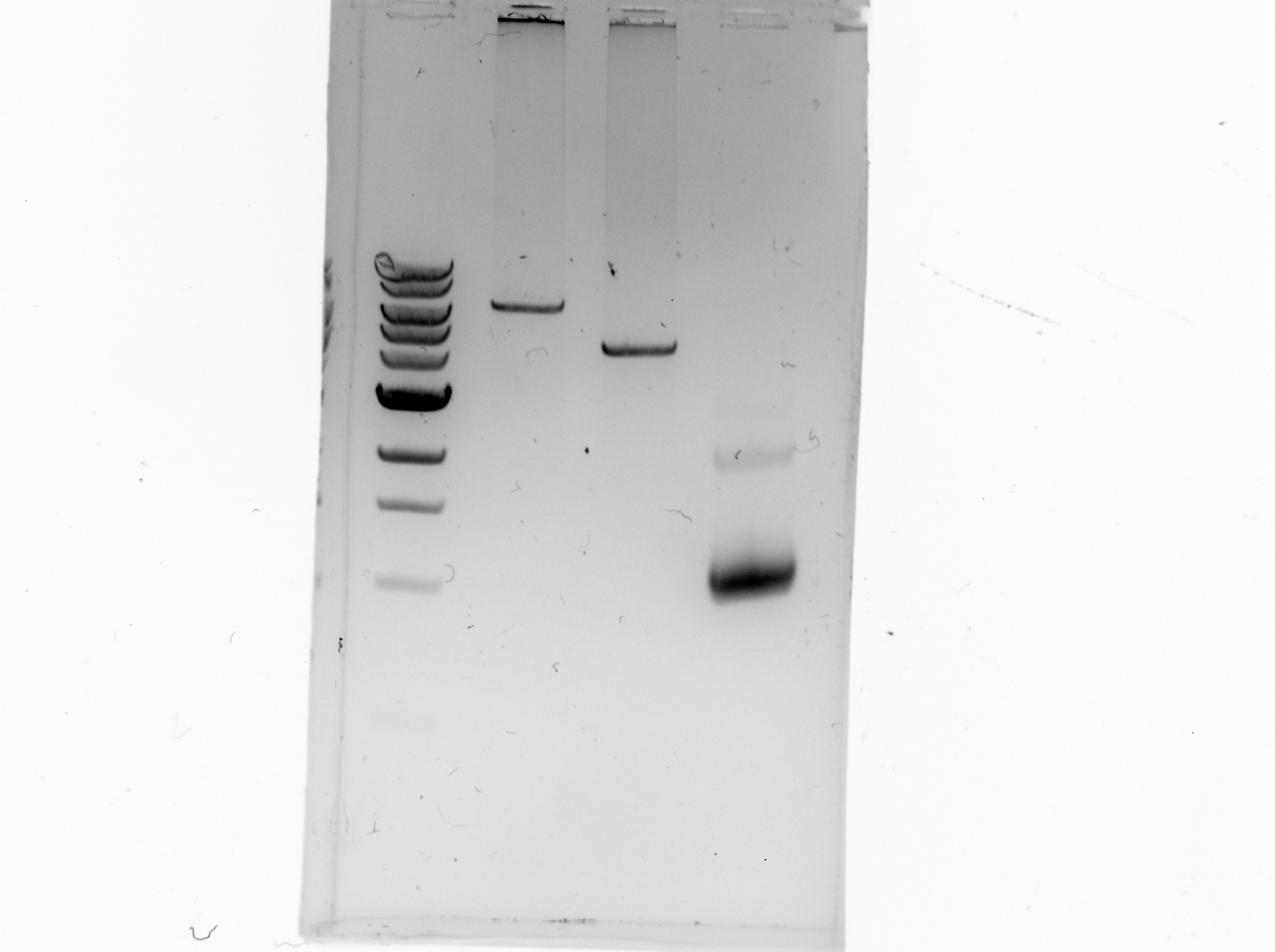

Supplement: Figure 5—figure supplement 2—source data 1. [file elife-89035-fig5-figsupp2-data1.zip › Figure 5-figure supplement 2-Source Data 1/Figure 5-figure supplement 2E_gel uncropped.tif]

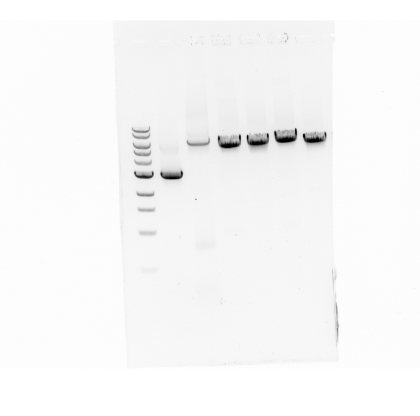

Supplement: Figure 5—figure supplement 2—source data 1. [file elife-89035-fig5-figsupp2-data1.zip › Figure 5-figure supplement 2-Source Data 1/Figure 5-figure supplement 2B_gel uncropped_2.tif]

Figure 5-figure supplement 2

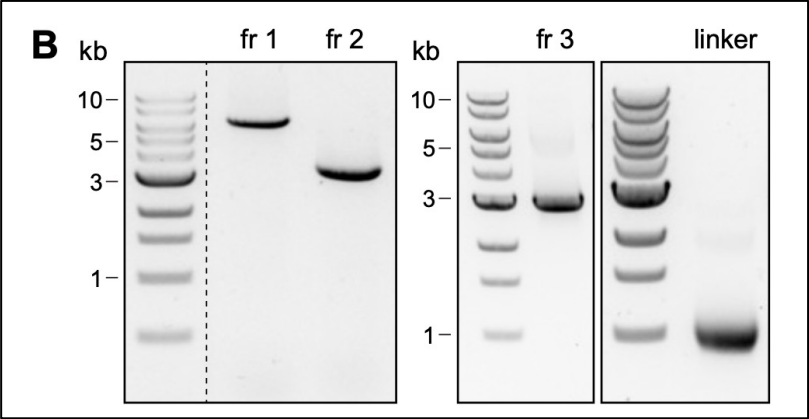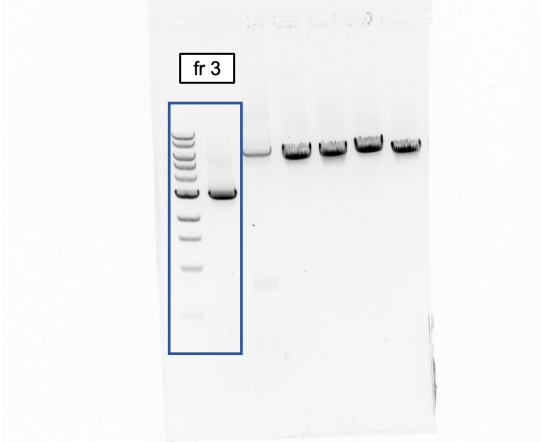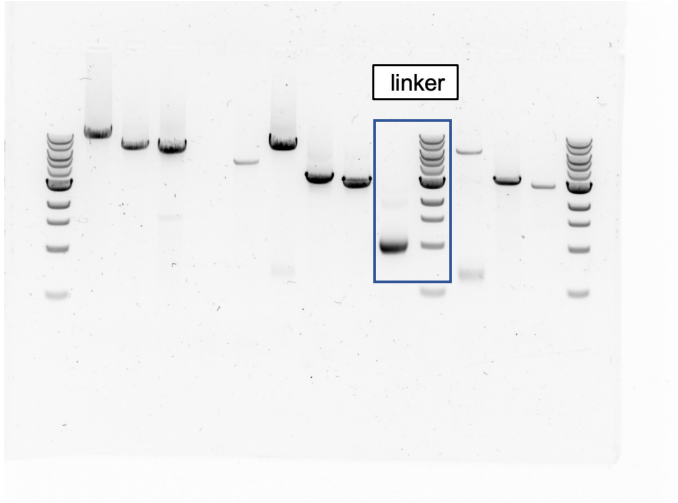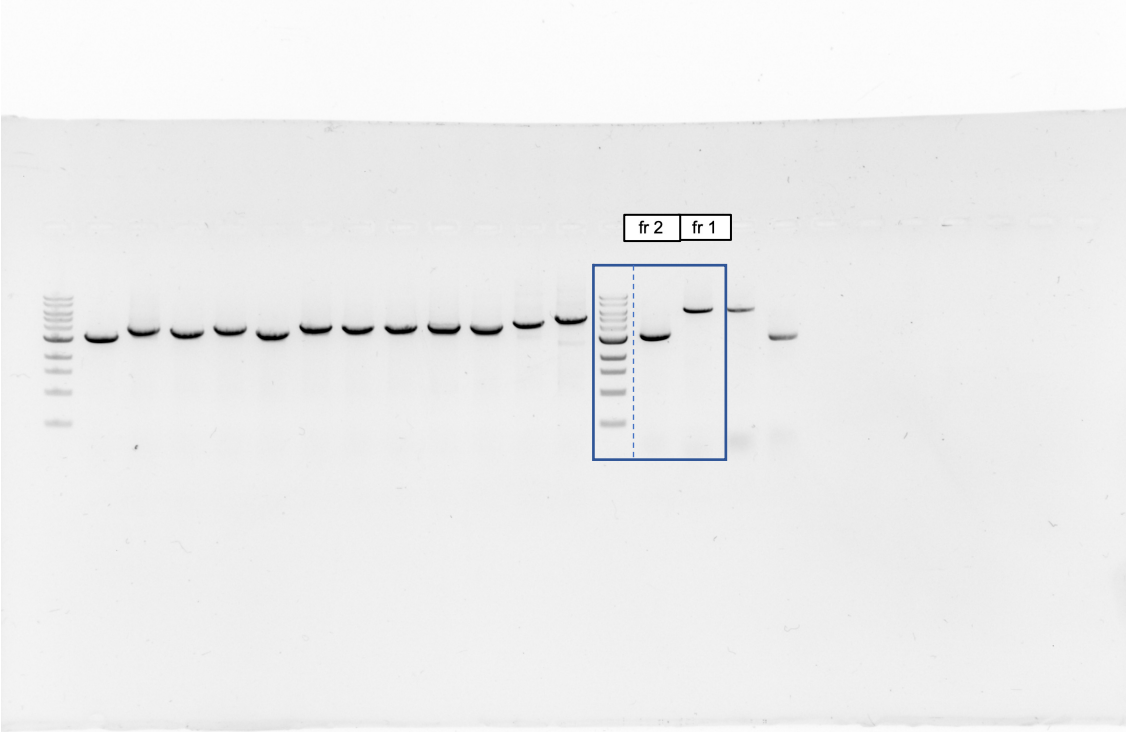

Figure 5-figure supplement 2

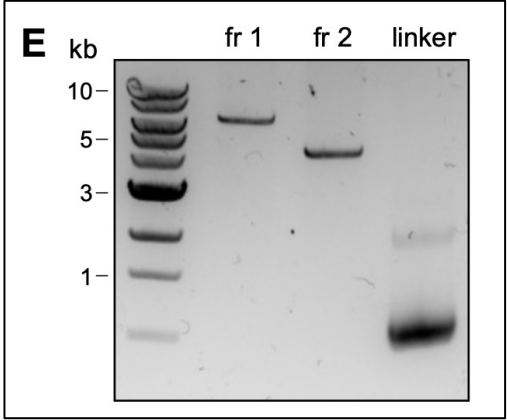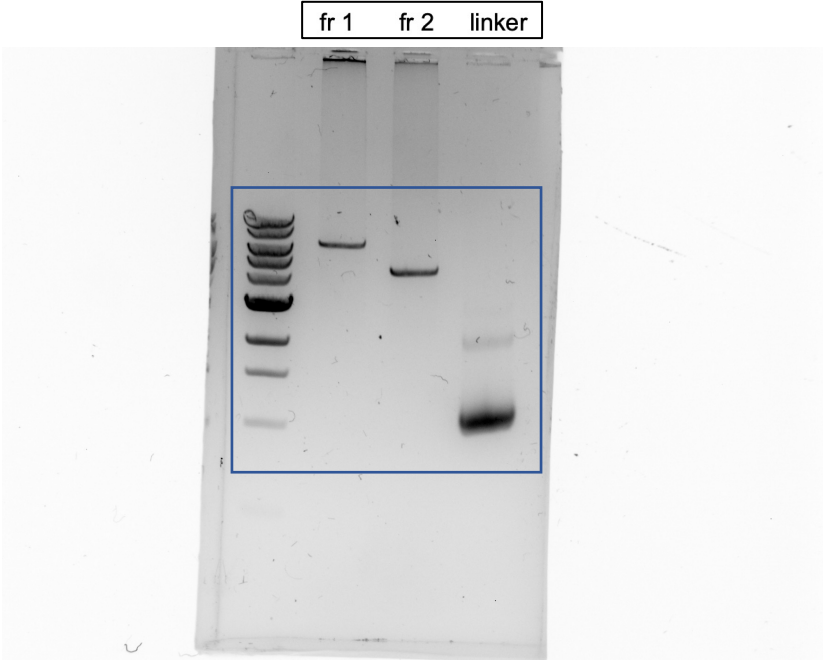

Supplement: Figure 5—figure supplement 2—source data 1. [file elife-89035-fig5-figsupp2-data1.zip › Figure 5-figure supplement 2-Source Data 1/Figure 5-figure supplement 2-Source Data 1.pdf]

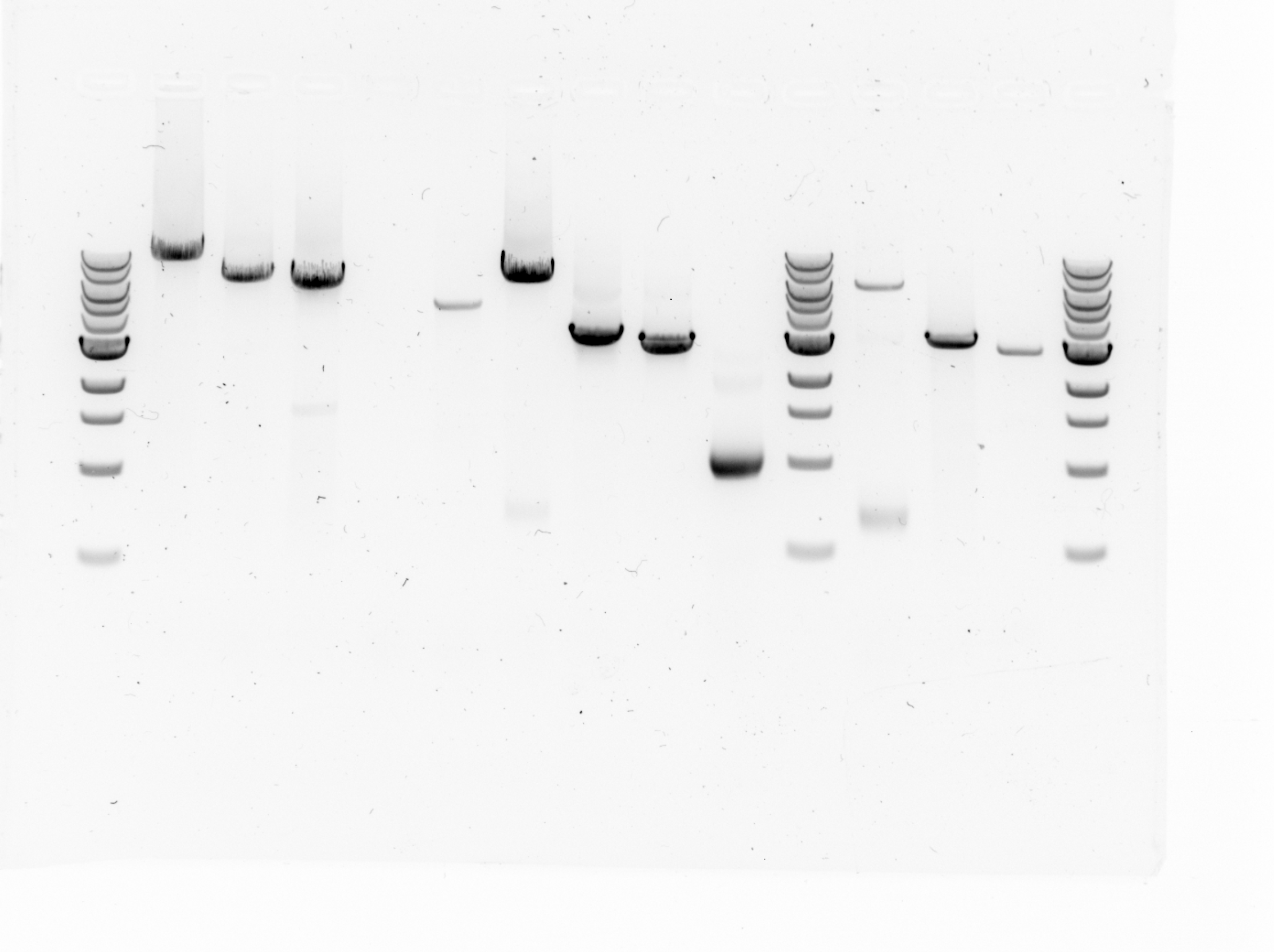

Supplement: Figure 5—figure supplement 2—source data 1. [file elife-89035-fig5-figsupp2-data1.zip › Figure 5-figure supplement 2-Source Data 1/Figure 5-figure supplement 2B_gel uncropped_3.tif]
