# Supplementary material for: Rapid cloning-free mutagenesis of new SARS-CoV-2 variants using a novel reverse genetics platform
Source: eLife. 2023 Nov 21;12:RP89035. doi: 10.7554/eLife.89035 (PMC10662946; doi:10.7554/eLife.89035)
Supplement: Supplementary file 1. — Vero E6 cells were infected with the indicated recombinant virus and pictures were taken 3 d post-infection. Of note, the virus was not titrated and the development of CPE is not quantitative. Scale bar represents 100 μm. [file elife-89035-supp1.pdf]

Supplementary File 1. Infectivity assessment of recombinant virus on Vero E6 cells.

Number of fragments	Transfection method	Name	Cell line transfected	3 days post infection on Vero E6	Description
-	-	Vero E6 negative control	-	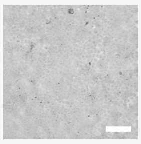	Uninfected Vero E6 cells.
8	electroporation	rCoV2-8fr	HEK293T	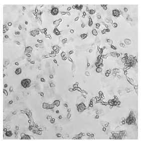	Rescue from eight fragments.
4	electroporation	rCoV2-4fr	HEK293T	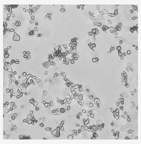	Rescue from four fragments.
4	electroporation	rCoV2-293	HEK293	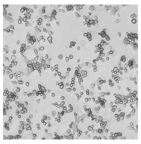	Rescue from HEK293.
4	electroporation	rCoV2-CHO	CHO	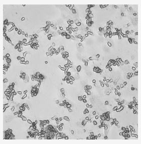	Rescue from CHO.
4	electroporation	rCoV2-BHK	BHK-21	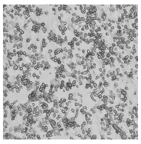	Rescue from BHK-21.
4	electroporation	rCoV2-A549	A549-ACE2	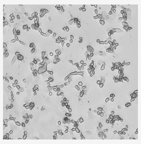	Rescue from A549-ACE2.
4	jetPRIME	rCoV2-jP	HEK293T	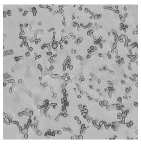	Rescue after transfection with jetPRIME.
4	Lipofectamine-3000	rCoV2-3000	HEK293T	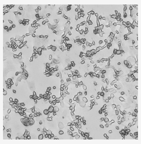	Rescue after transfection with Lipofectamine-3000.
4	Lipofectamine-LTX	rCoV2-LTX	HEK293T	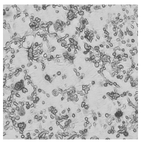	Rescue after transfection with Lipofectamine-LTX.

5	electroporation	rCoV2-500bp	HEK293T	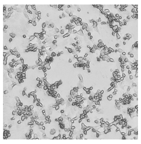	Rescue including one fragment to be only 500 bp in length.
5	electroporation	Wuhan$^{BA.1\ S}$	HEK293T	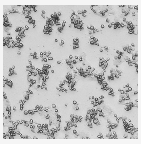	Chimeric virus, Omicron BA.1 S gene in the background of Wuhan.
5	electroporation	Wuhan$^{BA.5\ S}$	HEK293T	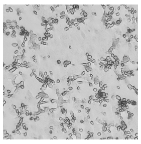	Chimeric virus, Omicron BA.5 S gene in the background of Wuhan.
5	electroporation	rCoV2-N501Y	HEK293T	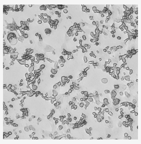	Direct mutagenesis, incorporation of N501Y substitution in the S gene.
5	electroporation	rCoV2-G614D	HEK293T	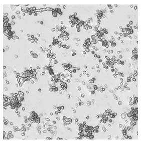	Direct mutagenesis, incorporation of G614D substitution in the S gene.
5	electroporation	rCoV2-ΔORF3a	HEK293T	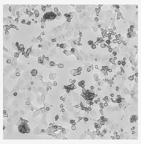	Direct mutagenesis, deletion of ORF3a.
5	electroporation	rCoV2-ORF8-FLAG	HEK293T	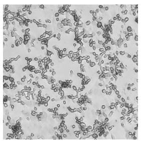	Direct mutagenesis, incorporation of a 3x-FLAG tag at the C-terminal of ORF8.
9	electroporation	rCoV2-link-Wuhan	HEK293T	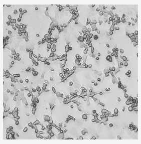	Rescue from RNA directly using the linker fragment.
9	electroporation	rCoV2-link-Omicron BA.5$^{BA.1\ S}$	HEK293T	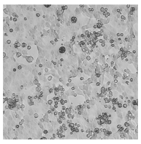	Rescue of chimeric virus from RNA directly using the linker fragment. Omicron BA.1 S gene in the background of Omicron BA.5.
9	electroporation	rCoV2-link-Omicron BA.1$^{BA.5\ S}$	HEK293T	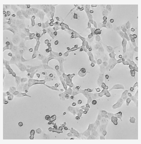	Rescue of chimeric virus from RNA directly using the linker fragment. Omicron BA.5 S gene in the background of Omicron BA.1.
9	electroporation	rCoV2-link-Omicron BA.5$^{Wuhan\ S}$	HEK293T	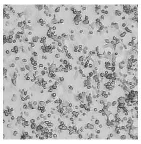	Rescue of chimeric virus from RNA directly using the linker fragment. Wuhan S gene in the background of Omicron BA.5.
